# Breakthrough on radon individual monitoring and protecting miners by novel dual-function monitor on respirator

**DOI:** 10.1038/s41598-023-48092-7

**Published:** 2023-11-27

**Authors:** M. Sohrabi, P. Khodaee

**Affiliations:** Health Physics and Dosimetry Research Laboratory, Department of Energy Engineering and Physics, Amirkabir University of Technology, Tehran, Iran

**Keywords:** Experimental particle physics, Physics, Applied physics, Particle physics

## Abstract

Breakthrough is made on inventing, producing and applying novel dual-function passive individual radon monitor in canister on respirators for radon inhalation monitoring and protecting individuals in particular miners. The rationale in this invention is having individual monitors for determining actual naturally inhaled radon by individuals and protection against particulates in one device. The monitor comprises two passive polycarbonate track detectors (PCTD); one near canister orifice (PCTD/bare), and one under activated carbon fabric (PCTD/ACF) to detect alphas of radon adsorbed on ACF carbon active sites. The PCTD/ACF detects radon with 12.7 times more sensitivity than PCTD/bare; called “Forced Amplification Factor (FAF)”. Monitors were successfully operated and calibrated in laboratory radon chamber using low suction rate pump resembling human natural inhalation. The performance of monitor showed high promises for radon individual monitoring and protection. This novel development also opens new horizons for fundamental and practical scientific research to further upgrade the monitor.

## Introduction

Protection against health hazards of human exposure to radon is a matter of serious concern to workers, public, regulatory authorities, and international organizations^1–6^. A recent document by the International Atomic Energy Agency (IAEA) focuses on occupational radiation protection in the uranium and processing industry in particular on the potential adverse health effects associated with uranium mining^6^. Also, miners have history of detectable exposure to radon and radioactive dust particles since centuries ago even in non-uranium mines where the first silver deposits were found in the Saxon Erzgebirge with potential radon of 50 kBq m^−3^ to 100 kBq m^−3^ in old mines, and 1 MBq m^−3^ where still 250 miners are permanently engaged in rehabilitation work to protect miners and public^7^.

A worldwide review of radon exposure in non-uranium mines such as in coal and metal mines provides detailed information on radon exposure levels and on active and passive radon monitoring devices used in different countries^8^. However, no information on the protection against other gases and dust radioactive particulates have been provided. While radon is subject to monitoring in uranium mines, it is not yet concerned as regulatory control need issue for non-uranium mines by the regulatory authorities. In fact, some potential wide range adverse health effects of miners in uranium and non-uranium mining are the same from which protracted exposure to radon gas and progeny represent the highest radiation-related cancer risk^1,6^.

Some active devices AlphaGUARD, PRASSI meter, KF606 and RAD7, etc. are used for workplace monitoring in uranium and non-uranium mines^8^, as well as other radon environments. As regards individual radon monitoring, passive polymer track detectors CR-39, LR-115, CN-85, polycarbonate track detectors (PCTD), etc. are commonly encapsulated in a small cup and used on the chest or helmet of a miner for monitoring periods up to 6 months or may be longer or on the wall of houses for radon resident monitoring. Such passive radon monitors have been under development for many decades. Such developent historically includes radon film badge with nuclear track emulsion^9^, or most widely used polymer track detectors such as cellulose nitrate^10^, LR-115^12,13^; CR-39^14,15^; PCTD^16–21^, Makrofol and activated charcoal^22^; CR-39 with activated carbon fabric (ACF) (CR-39/ACF)^23^, and PCTD/ACF radon monitors have been well studied and used^20,21^. Some researchers have even applied miniature pumps in a small pen-type monitor to collect air radon/progency and particulates on a filter in contact with a passive track detector to enhance the response^10,24^.

While passive polymer track detectors fulfil to some extent the requirements for individual and environmental radon monitoring indoors and outdoors, these monitors are usually required to be worn by miners or individuals at least for 3 months even when sensitive CR-39 detectors are used^7^. The sensitivity of such detectors was enhanced by applying an external adsorptive material such as activated charcoal powder^22,25^ or CR-39 with activated carbon fabric (CR-39/ACF) (ACF 1000 from Kareray Chemical Company, Ltd) as introduced as a radon-film-badge with ability to adsorb radon on its carbon active sites^23^ and PCTD/ACF introduced as twin badges^20,26^. Recently, by applying PCTD/ACF, some basic phenomena and a new multi-function individual/environmental radon badge was introduced with 1.5 times higher sensitivity than that of PCTD/bare as studied for radon concentrations up to 3 kBq m^−3^, based on which the individual monitoring period was reduced by 1.5 times^20,26^.

The passive radon monitors developed so far by us and others are usually worn on the chest or helmet of a miner to represent radon inhaled by miners with high uncertainties. Such methods of course will not provide the actual dose received by an individual. Therefore, in order to improve, simplify, and address the many deficiencies of radon individual monitoring and protection of individuals in particular miners, a novel dual-function passive radon individual monitor on a respirator (hereafter called monitor) was invented and studied in detail. The monitor worn directly on face of an individual can simultaneously; (1) monitor and determine the level of radon directly inhaled from nose, as well as inherently filters some radon gas by adsorption and some radon progeny and some dust particulates through the ACF filter when air is naturally inhaded by an individual or by a low flow rate pump. Therefore, it is the pupose of this paper to:

1. Introduce a novel “dual-function passive radon individual monitor on respirator” for monitoring directly real radon inhaled by an individual and simultaneously filtering some radon gas/progeny and some dust particulates,
2. Present details on design of the monitor components as invented, machine-made and developed using two independent passive PCTD/bare and PCTD/ACF detectors,
3. Study the detection responses of the monitor in the laboratory radon chamber with low air suction rates resembling an individuals’s direct natural air inhalation, and
4. Discuss the advantages and disadvanteges of the methods used and success in performance of the novel monitor invented and proposing additional extended research studies on some monitor parameters which are under our priority list to be performed.

### Radon

Radon is a general term used for its three major radioisotopes ^222^Rn, ^220^Rn and ^219^Rn emanated from three premordial long chain series with very long half lives (T_1/2_) respectively headed by ^238^U, ^232^Th and ^235^U. In fact, thorium and uranium are two of the most common abundant radioactive elements on earth. ^222^Rn (T_1/2_ = 3.8 d), and ^220^Rn (T_1/2_ = 55.6 s), and especially ^222^Rn are of high natural environmental health hazards to workers and public due to being inert gases and generating high energy alpha particles. However, ^219^Rn is of less concern due to its very low abundent compared to ^222^Rn since ^235^U abundent to that of ^238^U is very low. Also ^220^Rn with its 55.6 s half life is of second importance since its half life is not high enough to penerate the walls and other thick materials of concern. Therefore, inhalation of ^222^Rn in general by public and in particular miners worldwide is the second cause of lung cancer after cigaret smoking^1,3–6^. ^222^Rn emits a 5.49 MeV alpha particle and is a gas product of ^226^Ra (T_1/2_ = 1620 y) which is itself decay product of ^238^U (T_1/2_ = 4.5 × 10^9^ y). ^222^Rn decays to a non-gasous ^218^Po (T_1/2_ = 3.05 m) which emits 5.99 MeV alpha paticles and ^214^Po (T_1/2_ = 0.164 ms) which emits 7.69 MeV alpha particles. ^218^Po and ^214^Po are non-gaseous charged atoms which can easily attach themselves to aeorosols in air and are of serious particulate health concern. Therefore, the term radon used hereafter concerns ^222^Rn.

### Novel dual-function passive individual radon monitor

A novel dual-function passive radon individual monitor was invented, machine-made and mounted on a 3M respirator as for monitoring directly an individual’s inhaled radon and protection.

The canister comprises of the radpn monitor with an external case machine-made from solid acrylonitrile–butadiene–styrene (ABS) plastic material. The cansiter when assembled tight encompasses the monitor with all the internal detectors and support components intact. The monitor applied two PCTDs to primarily demonstrate some basic phenomena and concepts; one PCTD/bare placed at 6 mm distance under the canister orifice for detecting directly alpha particles from radon and its progeny, and one PCTD/ACF placed in the middle which detects alpha particles from radon adsorbed on the ACF carbon active sites for enhancing the response. The ratio of the track densities of PCTD/ACF to PCTD/bare under no air suction introduced an amplification factor (AF)^20^. Under air suction or direct inhalation, a new factor is introduced as here called “forced amplication factor” (FAF), as is discussed later in this paper. The ACF layer was mounted on a polyethylene ring tight in place so that air sucked through the orifice can only pass through the ACF layer.

In the preliminary experiments, both PCTD/bare and PCTD/ACF were in place in the cansister in order to compare the detector responses either under no suction or under suction by inhalation or by a pump. However, in the final ideal design, only one PCTD/ACF is used by removing the PCTD/bare and its support components from the canister leaving only the PCTD/ACF. Experiments were also performed by using two similar canisters on a respirator.

**Figure 1:**
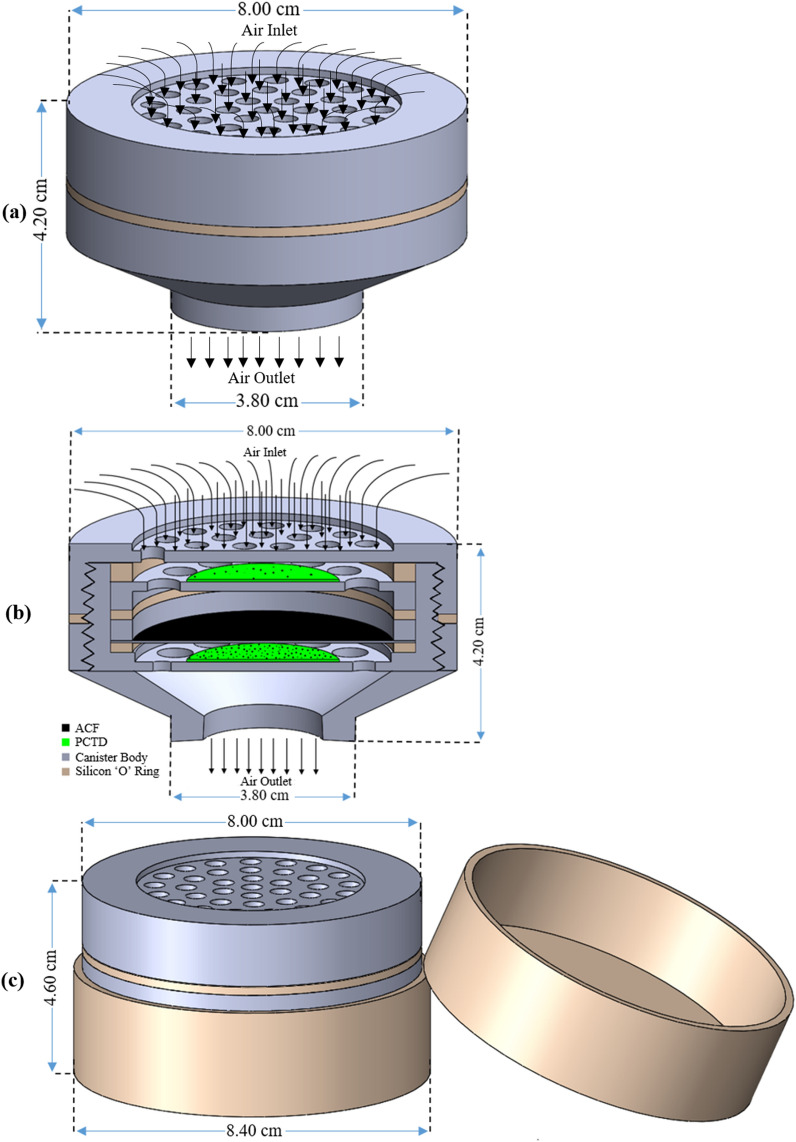
(**a**,**b**,**c**). Schematic design of the radon individual monitor in cansiter; (**a**) external view under suction, (**b**) cross sectional view, and (**c**) radon-tight silicon case in order to stop radon entering the canister when not in operation.

The Fig. 1a,b,c shows schematic design of the radon individual monitor in cansiter when air is sucked through its orifice including; (a) external view of the canister when under air suction, (b) cross sectional view of internal components, and (c) assembled canister with a silicon radon-tight case to stop radon entering the canister when not in use, the thickness of which needs to be optimized.

The Fig. 1c demonstrates symbolically the use of a silicon case over the canister when it is not worn by an individual. Presently, it is a common practice that radon individual monitors are placed in a radon-tight case after miners leave the work area^7^. This is based on the present radiation protection philosophy of the International Commission on Radiological Protection (ICRP)^2,27^, which defines a worker as an individual who gets exposed to ionizing radiation only occupationally during work with no consideration of non-occupational exposures of a worker as a member of public. Therefore, the silicon case of Fig. 1c is to meet the present ICRP requirements so that the cansiter is not exposed to radon when not in use, as is presently practiced^7^. However, the Universal Radiation Protection System (URPS) recently hypothetized by Sohrabi considers a radiation worker basically also as member of public who additionally gets exposed to ionizing radiation occupationally during work; i.e. a worker integrates effective occupational and non-occupational doses to be used in record keeping by the regulatory authority^28^. Therefore, in order to meet the criterion of the URPS hypothesis, there seems no need to use the silicon cap when the monitor is not in use after work^28^. The protocol for managing the monitors outside the working hours is under development.

**Figure 2:**
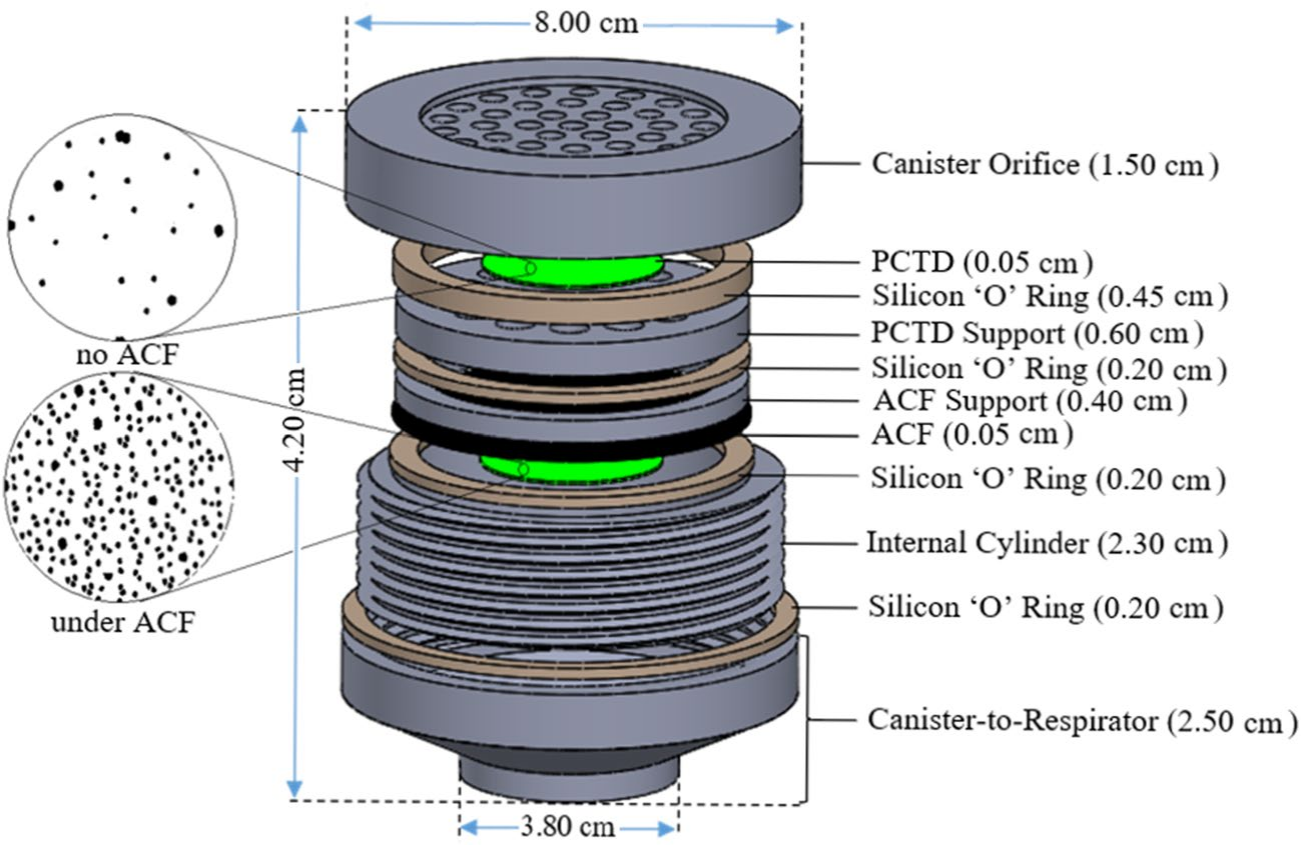
The canister components; (**a**) detailed internal components in particular focused on the two passive PCTDs after ECE processing; one PCTD/bare at 6 mm distance under the canister orifice, and one PCTD/ACF in the middle with support components.

**Figure 3:**
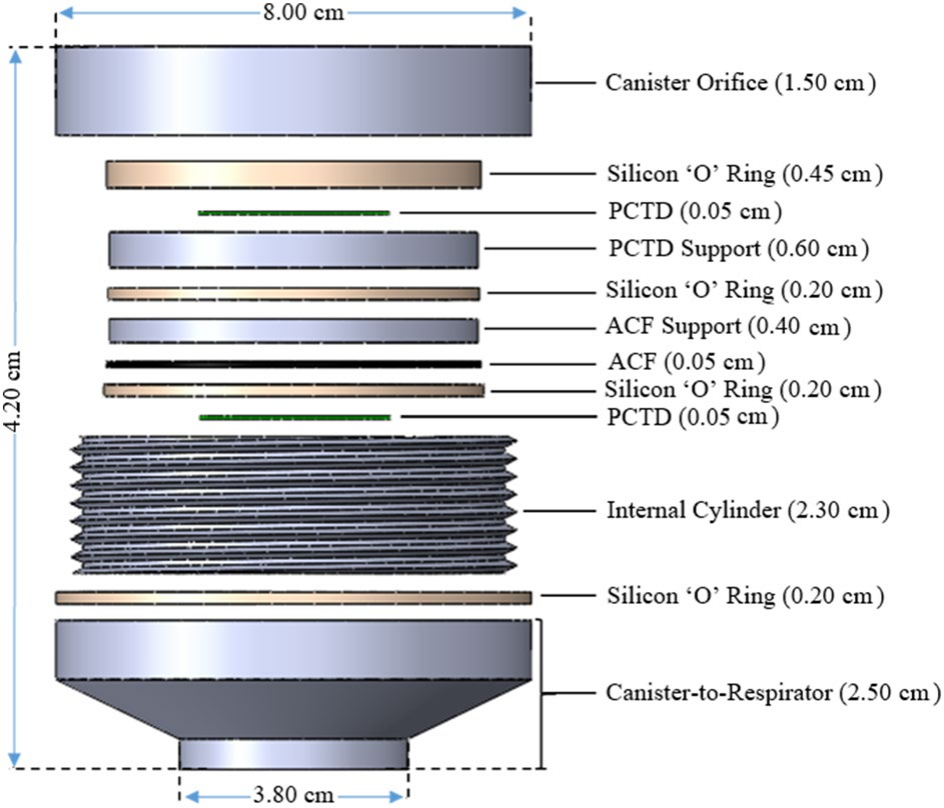
The cross sectional view of the intenal components of the caister.

The monitor encapsulates the monitor with several components as shown in Figure 2. It also holds two PCTDs as the main detectors, as explained above. The figure shows detailed components with dimensions in particular demonstrating the two PCTD/bare and PCTD/ACF detectors after the PCTDs have been exposed to radon alpha particles and processed by a triplet ECE chamber^29^, as will be discussed below. It is well observed that the ECE-processed alpha tracks in PCTD under ACF has much higher track density due to the adsobtion of radon in ACF cabon active sites than the bare ECE-processed PCTD. When the monitor in canister is well assembled together tight and mounted on respirator, then the individual plays the role of a natural pump and inhales air through the canister based on which the monitor operates as a passive direct individual radon monitor. Figure 3 also shows the cross sectional view of the intenal components of canister.

When air circulates through the canister has the only option to pass through the ACF filter. The ACF filter adsorbs a percentage of radon passed through it on its carbon active sites and simultaneously and “inherently” filters some radon progeny and dust particulates, the percentages of which have yet to be further studied. The PCTD/ACF then detects alpha particles from radon adsorbed on ACF carbon actibe sites. The PCTD/ACF will also amplifies alpha track density relative to that of the PCTD/bare. An amplification factor (AF), i.e. ratio of alpha track density of PCTD/ACF to that of PCTD/bare when no suction is applied has been recently introduced and studied by us^20,26^. Even at room environmental conditions with no forced air suction, the AF value is > 1 and remains about 1.5 up to a radon concentration 3 kBq.m^−3^ studied^20,26^. However, when air is sucked through the canister by force either by pump or naturally inhaled by an individual, the two PCTDs are exposed relatively to higher radon concentrations with more alpha tracks registered on them. This leads to a higher sensitivity ratio of the PCTD/ACF relative to PCTD/bare under forced air suction. This enhanced forced sensitivty ratio is introduced here as “forced amplification factor (FAF)” as formulated below:

$${\text{FAF}} = {\text{ D}}_{{{\text{ACF}}}} /{\text{D}}_{{{\text{bare}}}}$$

where:

D_ACF_ = alpha track density on PCTD under ACF layer, and.

D_bare_ = alpha track density of bare PCTD with no ACF.

**Figure 4:**
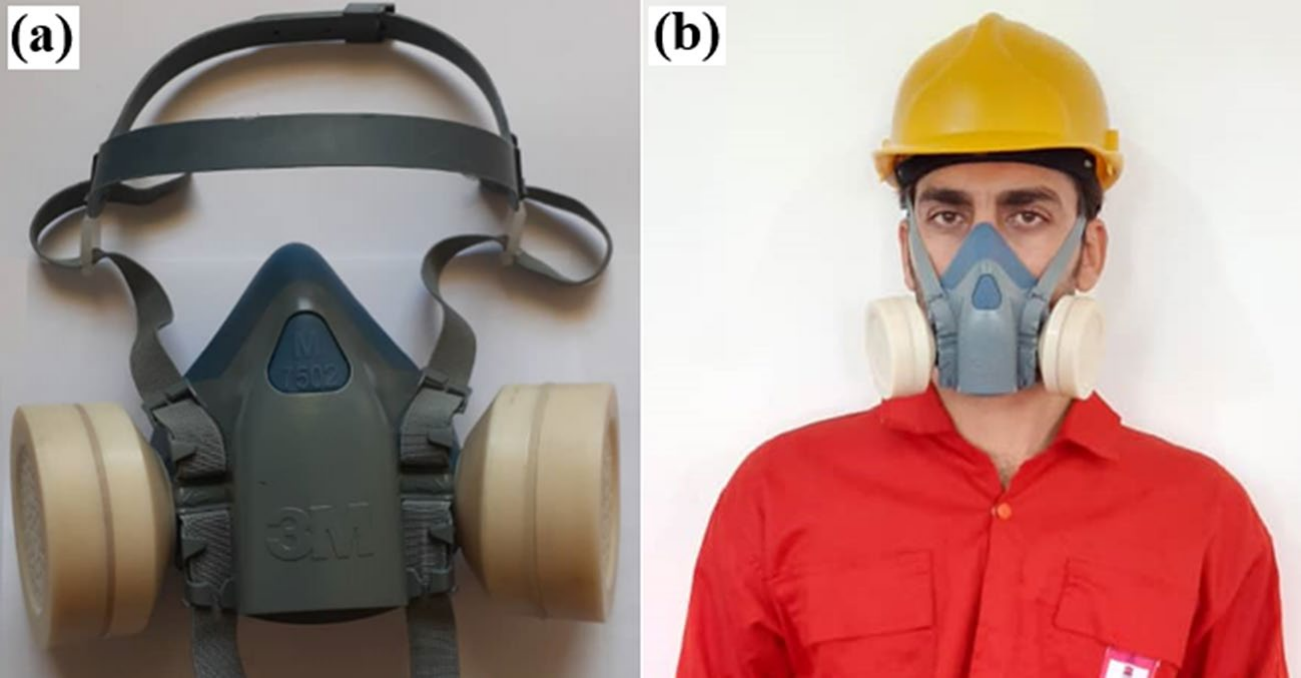
(**a**, **b**). Two dual-function individual radon monitoring cansiters mounted on a 3M respirator (**a**) and when worn by a representative miner (**b**) (Mr. Payam Khodaee the coauthor).

In order to study the performance of the monitors produced, either one or two monitors were mounted on a 3M respirator (from 3M Personal Protection Productive manufacturer) and used by a representative miner, as shown in Fig. 4a,b.

It should be noted that unlike active charcoal with large adsorptive surfaces which is highly affected by humidity and temperature^20,23^, the ACF is an adsorptive solid radiative fabric with a large surface-to-volume ratio which adsorbs radon concentrations much higher than that of the surrounding air^23^. As detailed before^20^, the ACF used in our studies is like a tissue made of “acrylic fabric” stabilized and then carbonized with stepwise sequential heat treatment followed by activation with steam^20,30^. The ACF layer with a low humidity sensitivity regains 1.0%–2.5% moisture under standard temperature and humidity conditions. Therefore it fulfils the requirement of being used in an open environment with possible moisture for a long period of time after proper calibration of the monitor under the conditions of the working environment of comcern. In another study, we have used the PCTD/ACF detector in high radioactive spring waters of Ramsar being high level natural radiation areas for determination of ^226^Ra and ^222^Rn under 100% moisture. The results being under preparation for publication are in good agreement with alpha spectrometry methods of the same water samples. Of course the responses may also depend on the type of ACF used. However, some recent studies have applied ACF of different types under different moisture and temperature conditions, which are elaborated in the discussion session below.

## Experiments and methods

### Polycarbonate track detectors (PCTD)

PCTDs when processed by an ECE method^29^, in particular under 50 Hz – HV electric field conditions, as recently studied by us, have been applied in many recent studies. Such novel studies include efficient monitoring of radon and progeny^20^; breakthough on discovery of 4π ion distribution in plasma focus devices by 4π ion impage processing of hydrogen (proton)^31,32^, deuterium, helium or heavier ions^31,32^; alpha particle detection in a wide energy range^21^; megasize (40 cm × 80 cm) radon monitoring and ion image processing^21^, novel miniature neutron dosimeter/spectrometer for neutron dosimetry^33^, and passive novel multi-directional neutron spectrometry^34^.

The PCTDs used in the canister are 3 cm × 3 cm cut from a 500 μm thick polycarbonate sheet (masked on both sides) which is available in different thicknesses in common polymer markets. The radon calibration responses of the PCTD/bare and PCTD/ACF have been recently made at the radon calibration facility of the Hirosaki University in Japan^20^.

The PCTDs after exposure to alpha particles of ^222^Rn and progeny were processed by ECE methods^29^. The 50 Hz– 2 kV field conditions were applied for 500 µm thick PCTDs in optimized mixture of potassium hydroxide (15g KOH), ethanol (40g C_2_H_5_OH) and water (45g H_2_O) (PEW solution) at 26 ± 1 °C for 6 hours^20^. After the ECE processing, alpha particle tracks on PCTDs appear quite large observable even by the unaided eyes, as shown in Fig. 2a,b. The alpha particle tracks have been quantified by counting them under a Vikon light microscope usually at low magnifications (× 40) or computer softwares.

### Monitor exposure to radon

The monitor exposure studies were performed in the radon exposure chamber of our Health Physics and Dosimetry Research Laboratory. The radon source used in the radon chamber is ^226^Ra containing soil obtained from the high background radiation areas of Ramsar^19,20^. Radon concentration in the radon chamber was determined by PCTD/bare and PCTD/ACF individual/environmental radon monitors recently calibrated at the radon calibration chamber facility of the Hirosaki University in Japan^20^. The internal air of the laboratory radon chamer can be sucked out from its external outlets of the radon chamber, when necessary.

A low flow rate HSP pump with a potential variable flow rates of 0 to 24 L per minute of air was used in all experiements. The two PCTD/bare and PCTD/ACF detectors in the monitor were exposed by sucking air through by a low flow rate of 10 L per minute to resemble the breathing rate of a miner for exposure durations 0.5, 1.0, 2.0, 4.0, 6.0, 8.0 and 10 h. The air flow rate through the canister during all the experiments were kept constant.

### Experimental findings and results

**Figure 5:**
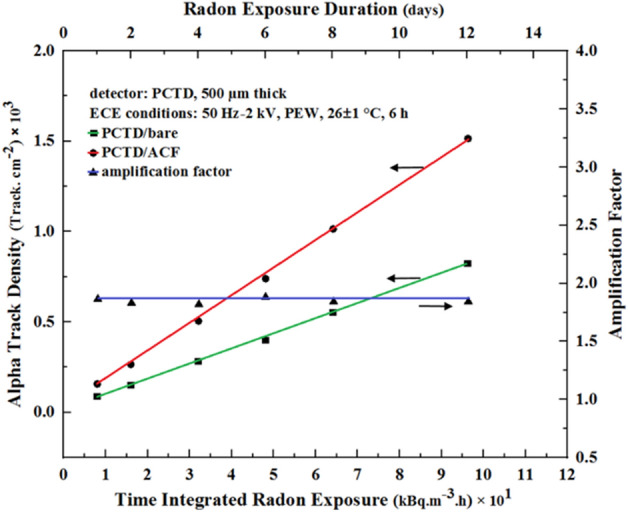
Alpha track density on PCTD/bare and PCTD/ACF detectors (left scale) and the AF values (right scale) versus time integrated radon exposure (lower scale) and radon exposure duration (upper scale).

In order to determine radon concentration in air passed through the monitor from the laboratory radon chamber, the monitor with two detectors in were first placed in the radon chamber environment with no air suction and were exposed for different durations. After radon exposure, the PCTDs were processed by ECE, track densities were determined, the AF values were calculated, and radon concentration was determined using the detector calibration factors obtained from the experiments made in the radon calibration chamber at the Hisrosaki University in Japan^20^. Figure 5 shows alpha track density (left scale) and the AF values (right scale) versus time integrated radon exposure (lower scale) and also versus radon exposure duration (upper scale).

As can be seen in Fig. 5, alpha track density responses of the two detectors are linear functions of time integrated radon exposure and radon exposue duration. Therefore, the AF response of the two detectors; i.e. the ratio of the track density of PCTD/ACF to that of PCTD/bare is flat with 1.7 value. Using the detector track densities and the track density-to-radon concentration calibration factors, radon concentration in the radon chamber was further determined.

**Figure 6:**
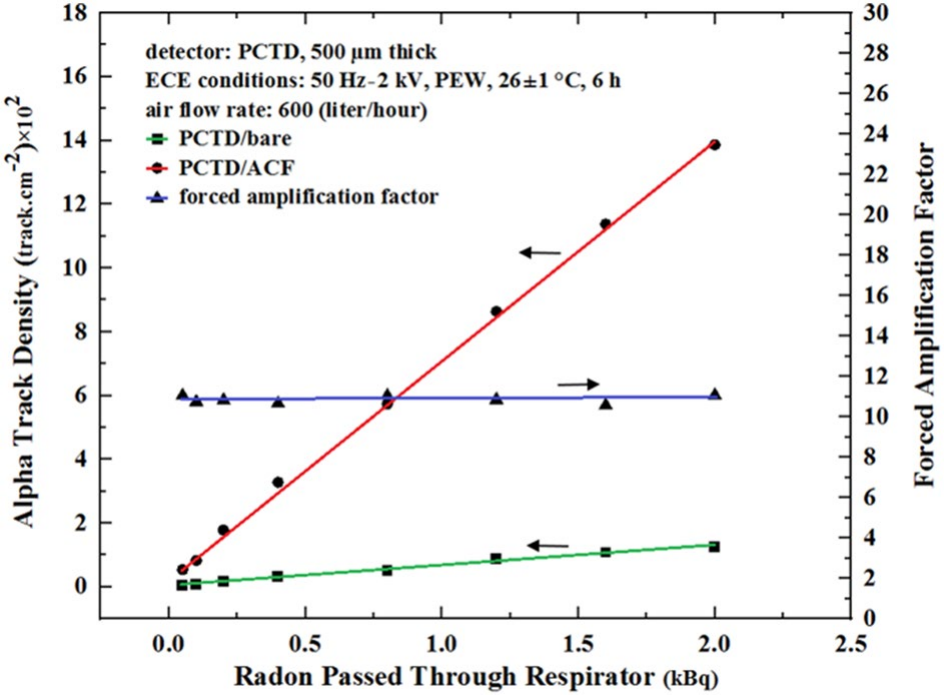
Alpha track density in PCTD/ACF and PCTD/bare (left scale) and FAF values (right scale) versus radon exposure duration using a flow rate of 600 L per hour.

In order to determine the characteristic response of the PCTD/ACF and PCTD/bare under forced air suction, which is the ideal situation in a radon field, the orifice of the canister was connected after being hermetically sealed to the low flow rate pump placed in the radon chamber with 600 L per hour flow rate for different exposure durations. After the radon exposure and PCTD ECE processing, alpha track densities and the FAF values for forced air suction were determined. Figure 6 shows alpha track density (left scale) and FAF values (right scale) versus radon exposure duration when radon air passed though the canister with a suction flow rate of 600 L per hour.

As can be seen in Fig. 6, alpha track density responses of PCTD/ACF and PCTD/bare under forced air suction versus radon exposue duration are also linear functions. Therefore, the FAF value response under radon flow rate of the pump is flat with a FAF value of 11.0 which is about 7 times higher than AF values when the monitor is under no air suction. The responses demonstrate the ACF carbon active sites role in enhancing detection efficiency. Table 1 compares microphotographs of alpha tracks on the PCTD/ACF and PCTD/bare after ECE processing for different air volumes and in turn for different radon concentrations passed through the monitor. The microphotographs also well demonstrate the role of ACF on enhancing PCTD/ACF response.

**Table 1:**
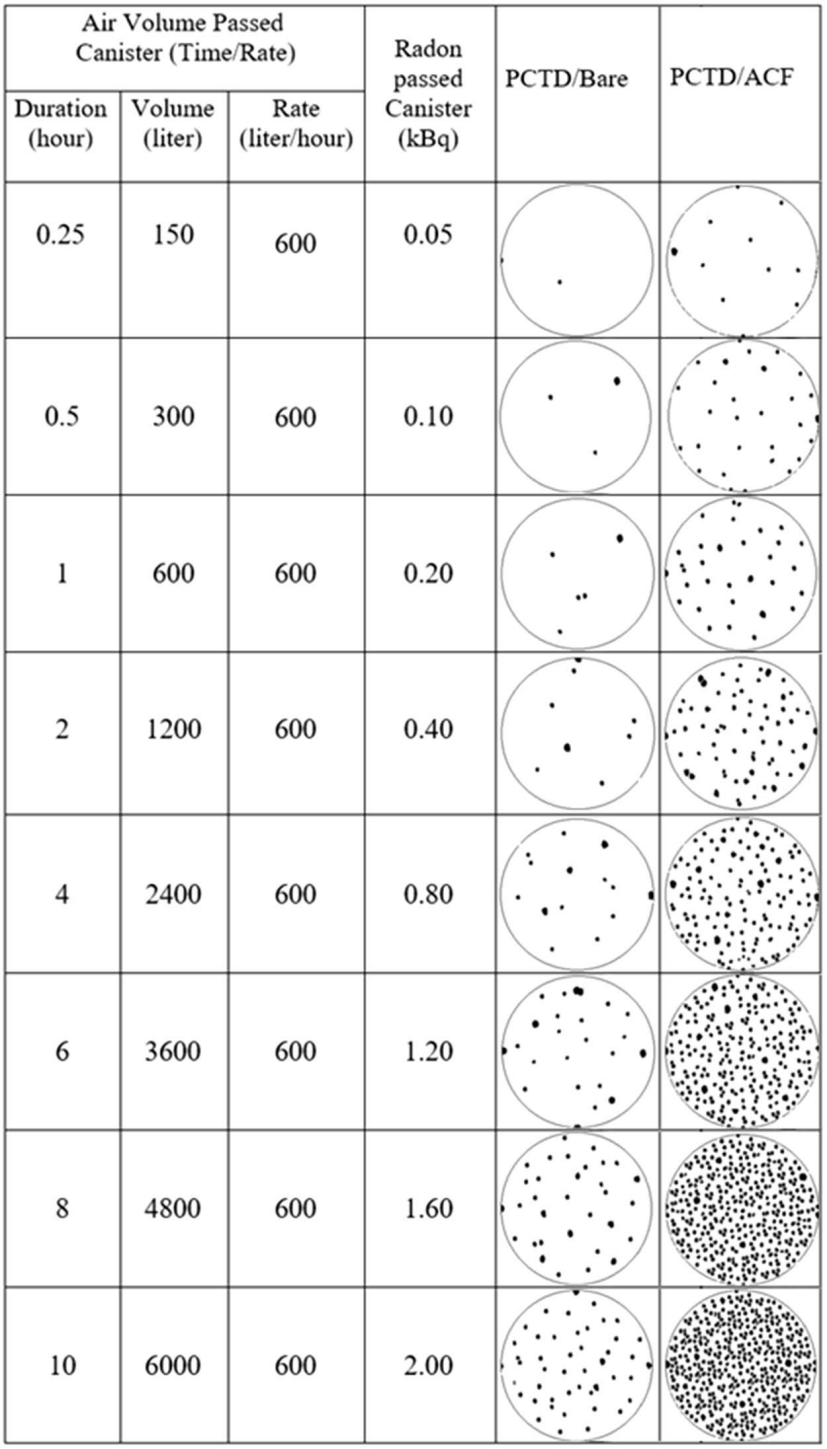
Microphotographs of alpha tracks on PCTD/ACF and PCTD/bare for different air volumes and radon concentrations passed through the monitor. The microphotographs also well demonstrate the ACF role on enhancing PCTD/ACF response; 11 times higher sensitivity than that of PCTD/bare.

**Figure 7:**
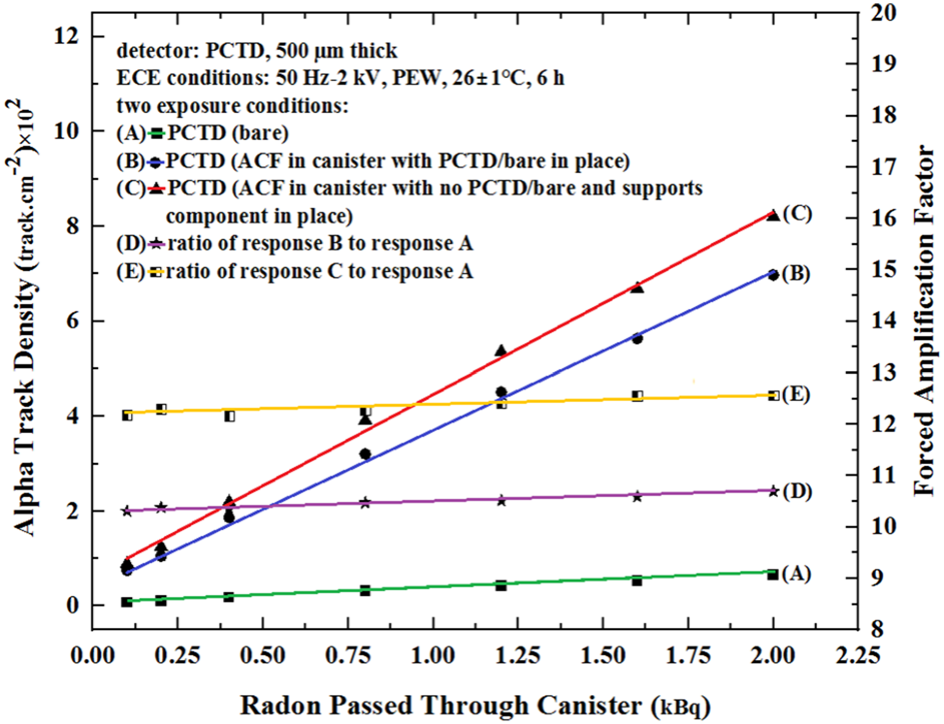
Comparison of alpha track density (left scale) and FAF values versus radon passed through the monitor exposing the PCTD/ACF when PCTD/bare is in place (response B), and when PCTD/bare and its support components have been removed from the monitor (response C).

The first monitor studied above, as shown in Fig. 5, has PCTD/ACF and PCTD/bare incorporated inside it for fundamental detector comparative response studies with no radon suction; i.e. when AF response of 1.7 was obtained. However, in the design and operation of a monitor for real applications, only one PCTD/ACF detector is enough to be used as a monitor. This simplifies the design, makes the monitor and in turn the canister thiner in thickness, and increases the PCTD/ACF sensitivity and FAF value, as seen in Fig. 7. This condition was studied by comparing PCTD/ACF sensitivity and FAF values versus radon passed through the monitor under two conditions; when PCTD/bare with support components are still in place in the monitor. The responses demonstrate that the FAF values were further inceased, as shown in Fig. 7.

As can be seen, just by removing the PCTD/bare with its support components, the PCTD/ACF sensitivity and in turn the FAF value was increased from 11 to 12.7 by about 20%. The basic experiments demonstrated the role of ACF on FAF values. However, more studies are underway in our laboratory to further enhance the PCTD/ACF efficiency and FAF value by applying different ACF types to also optimize the ACF diameter in particular for more breathing comfort of an individual, as discussed below.

**Figure 8:**
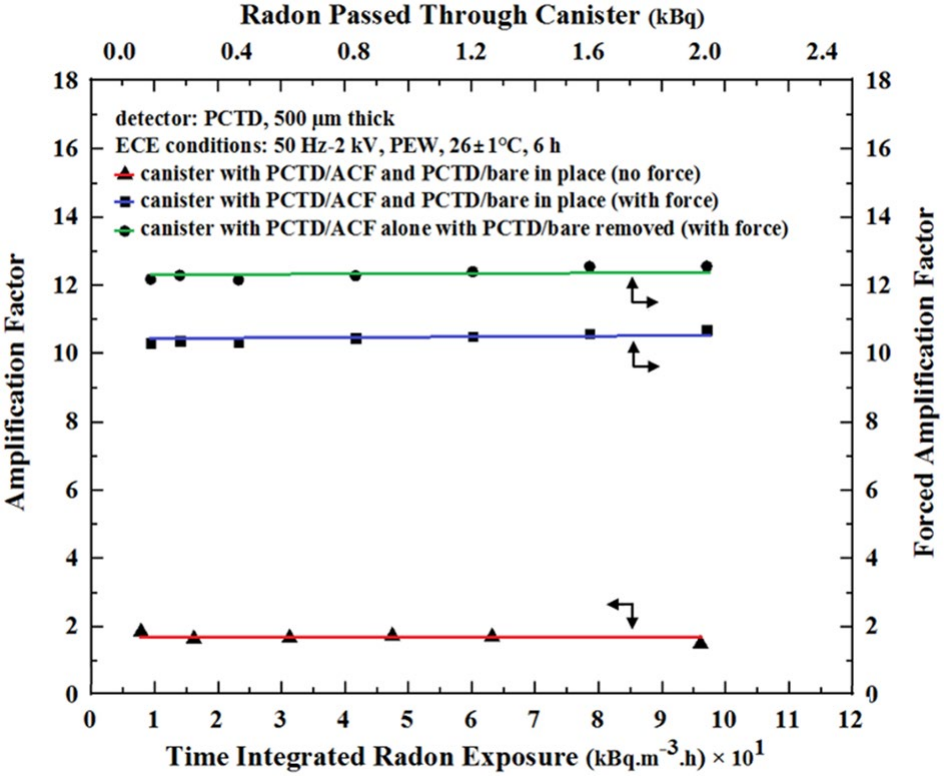
Comparison of AF response (left scale) versus time integrated radon exposure (scale below) and FAF value response (right scale) versus radon passed through the monitor (upper scale) exposing the PCTD/ACF when PCTD/bare and its support components are in place and when have been removed from the monitor.

Figure 8 compares the AF value response versus time integrated radon exposure and FAF value response versus radon passed through the monitor under different conditions applied. Figure 8 well demonstrates how the AF value of 1.7 with no external force has been increaded to 11 and then 12.7 values as explained above. The figure well compares the AF and FAF value responses.

**Figure 9:**
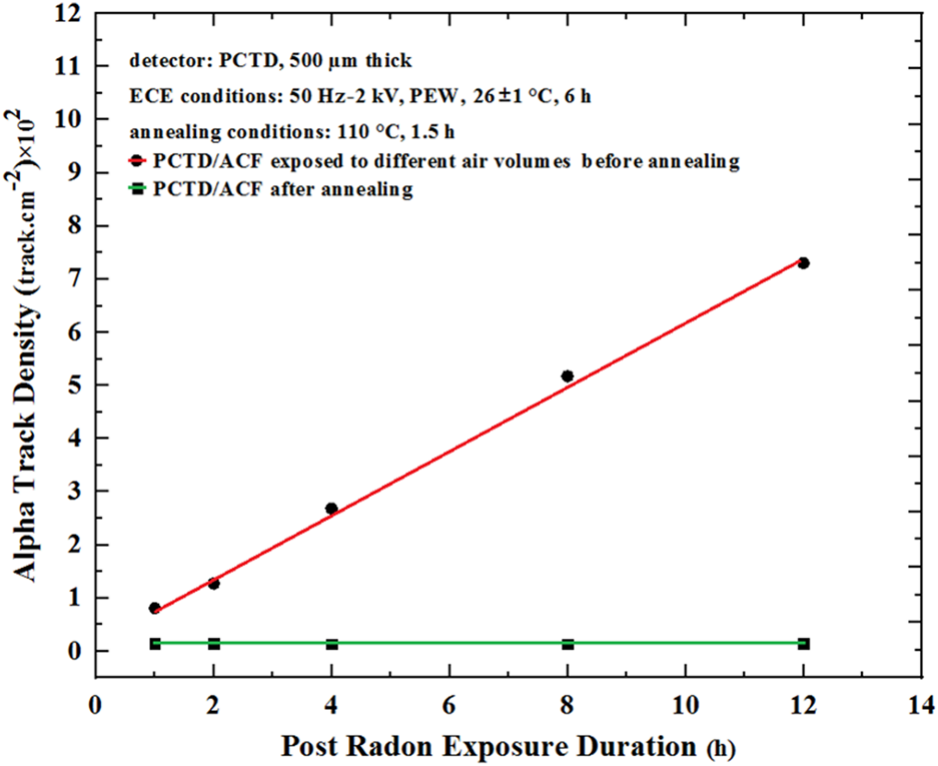
Comparison of alpha track density response versus post radon exposure duration before and after ACF annealing, proving further that the annealed ACFs released all radon adsorbed and the ACF can be reused again.

If an ACF filter is exposed to radon for a long time or in high radon concentration environments, its carbon active sites will be occupied by radon adsorbed and the sites no longer adsorb radon any more. However, our studies demonstrated that the ACF after use can be annealed at 110 °C for 1.5 h in an oven to restore the carbon activated sites^40^. This heat treatment anneals and releases all radon gases adsorbed on ACF carbon active sites as confirmed by alpha spectrometry of the ACF before and after annealing proceess^35^ and as further studied here. In order to further confirm the annealing effects, a number of PCTDs from exposed PCTD/ACF to radon under suction for different durations were prepared. Then ACFs were removed from the PCTDs, each ACF was placed in contact with a new PCTD and PCTD/ACF was kept in the laboratory for different durations. Under such conditions, the ACFs expose the PCTDs from radon adsorbed before and still exist on the ACF carbon active sites. Then ACFs were removed and annealed at 110 °C for 1.5 h in an oven. After annealing, each annealed ACF was placed again in contact with a new PCTD and kept for the same periods of time. Figure 9 shows responses of alpha track density versus post radon exposure duration before and after ACF annealing. This experiment proves further that the annealed ACFs can be reused again. However, for how many times the carbon active sites stay active and the ACFs can be reused after annealing, this is under further studies.

**Figure 10:**
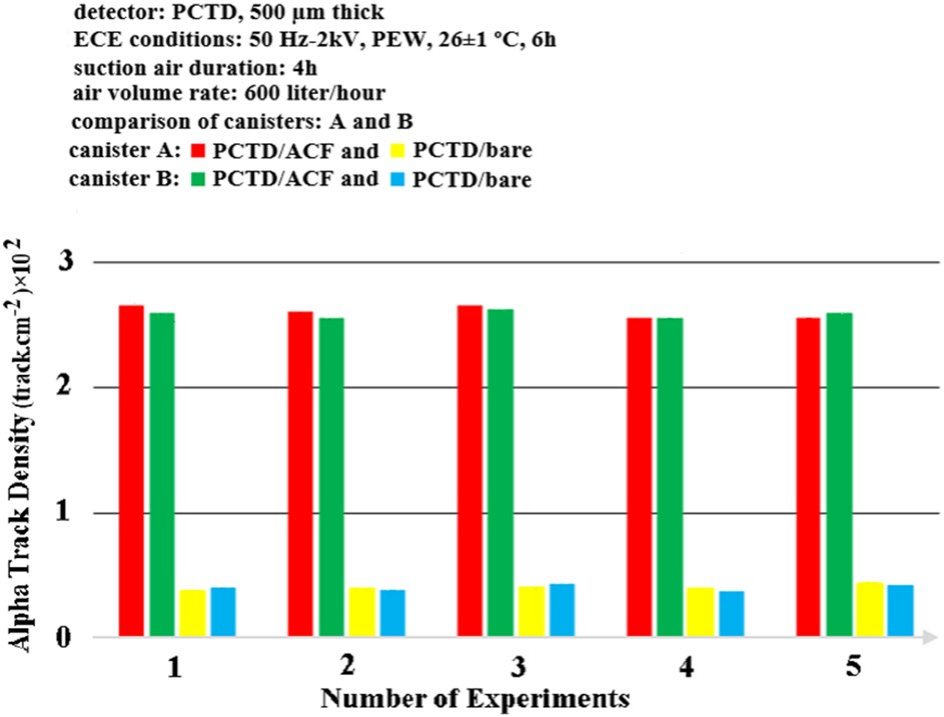
Comparison of the PCTD/ACF and PCTD/bare responses in each of the two monitors after 4 hour exposure duration through a radon air flow rate of 600 L per hour.

In order to specify the reproducibility of the monitors machine made, the responses of two monitors were compared by exposing them through low rate pump to radon. Figure 10 compares the responses of the PCTD/ACF and PCTD/bare in each of the two monitors for 4 h duration through a radon air flow rate of 600 L per hour. They were also processed under similar ECE processing comditions.

## Discussion

Miners in uranium and non-uranium mines and other radon environments have always been exposed to radon gas and progeny, and dust particulates. Miners in uranium mines are required by law to be equipped with safety and protection devices and methods for radon exposure and particulate monitoring and protection. Such conditions as mentioned in the introduction seem are of scientific intersest and for regulatory control in non-uranium mines.

In order to dermine radon concentration inhaled by a miner for calculating the effective radon dose, a miner is commonly monitored by an individual radon monitor such as CR-39 in a cup worn on the coveralls or helmets for a duration of at least 3 to even 6 months^7,8^. In parallel, the miners wear also disposable facepieces or reusable respirators to be protected from inhaling hazardous mine gases and particulates. The current miner’s monitoring programs and protection devices may have some deficiencies due to unavaibility of a device to monitor radon directly inhaled by the mouth. Some of the deficiencies may include:

1. In current practice, a radon individual monitor is commonly placed over miner’s coverall or on safety helmet which is located at a relatively long distance from real individual inhalation by mouth and the actual radon inhaled cannot be precisely determined. In fact, breathing is cyclic and not contninous while the detector on the chest detects radon in a continuous manner which causes an additional error. Also the radiation safety officer cannot be informed in time on the monitoring results.
2. CR-39 detectors of dosimetry grade commonly used are relatively at high cost, not available to researchers or service providers in many countries, available only in limited sizes, breakable like glass, should be cut like glass with care, vary in terms of sensitivity and background track density even from bach to batch, and requiring high temperature (e.g. 70 – 90 °C) chemical processing of alpha tracks.
3. The miners in order to be protected from hazardous gases, dust radioactive particulates, etc. may also be provided with disposable or reusable respirators. A miner then should use at least two separate protective devices; i.e. an individual radon monitor and a protective respirator separately.

In order to address the many deficiencies of the above-stated monitoring and protection devices and methods, a novel dual-function radon individual monitor was inevented, machine made and studied in detail. This individual radon monitor provides a number of advanteges such as:

1. It kills two birds with one stone; i.e. it itegrates the functions of the radon individual monitor and protection device to protect a miner from radon gas and particulates into one monitor which operates directly by natural inhalation rate of a miner. The monitor also inherently stops paticulates wnd radon gas by adsorption the efficiency of which depends on particulate size, type of ACF used, moisture, etc. The efficiency and its promotion are planned to be specifically studied in a carefully-designed project in our laboratory.
2. It reduces the monitoring duration many times based on the FAF values obtained. In our previous invention of novel multi-function PCTD/ACF individual/environmental radon twin badges^20^, and also in this study, it was proved that by using PCTD/ACF with no forced air into the canister, the AF value is about 1.7 times that of PCTD/bare which reduces the monitoring duration by approximately 1.5 times for example from 3 to 2 months. However, by pumping or inhaling radon gas through the monitor, a FAF value of 11 times was obtained with PCTD/bare and its components yet in the monitor, and 12.7 value (Fig. 8) when they are removed; i.e. the sensitivity of detection with PCTD/ACF is 12.7 times higher than of PCTD/bare when no air is inhaled by an individual or by sir suction. This FAF value decreases the monitoring duration significantly which should be optimized at any conditions applied. This FAF value is expected to be further increased when all basic parameters are optimized, the best type of ACF with optimum size is slected, etc.
3. Other than miner monitoring, the monitor can also be widely used for individual montoring of workplaces and indoors of houses. The common practice for determining resident’s effective doses is using polymer track detectors such as CR-39 or PCTD inside cups mounted on the wall of different rooms based on which the effective dose is estimated. Sometimes workplace monitoring is also applied. Such data have been used for epidemiological studies of pulic. This is far from the real situation since the individual moves around the house and the effective dose obtained from the wall monitors s very high uncertainty^18,19^. In particular, as also stated above, the detection by the wall monitors is continuous while breathing by an individual is cylic. Therefore, for epidemiologic studies. one person in the house might wear the monitor mask for example for a week to determine the actual inhaled effective dose.
4. The PCTD detector has many advantages over CR-39 such a low cost; availability in large sizes, well masked on both sides to prevent scratches, and in different thicknesses, and can be purchased even in common polymer markets. Different suppliers like Bayer Company provide high quality poycarboate sheets which can be well processed by low frequency electric field ECE conditions (e.g. 50 Hz–2 kV) at room temperature (e.g. 26 °C); observation of tracks by even the unaided eyes^16^, thus requiring simple track counting methods under a low magnification light microscope or by computer softwares; moisture and temperature resistance at different environmental conditions; etc. The ACF filter can be simply annealed and reused, as discussed above. However, for how many times the carbon active sites can stay active and ACF can be reused after annealing, this is under further studies.
5. As discussed above, the ACF due to its tissue texture nature is expected not to be too dependent on moisture, as discussed in the text before; This dependency has yet to be determined in anothed project. However, a recent study by comparing two types of ACF, namely ACC-5092–10 and ACC-5092–20, with different adsorbed water content from zero to saturated level^36^, demonstrated that radon adsorption in ACC-5092–10 smoothly and monotonically decreases with the increase of adsorbed water and at saturation level it is lower by a factor of 2.5 as compared to the fully dehydrated material. On the other hand, in ACC-5092–20 a clear break point was observed at water content of about 20% where radon adsorption sharply drops by a factor of 15. Therefore, ACC-5092–10 was recommended as a material that keeps its high radon adsorption ability even when saturated with water to be used for sensitive long-term ^222^Rn measurements even at high levels of humidity^36^. However, the moisture dependence of the radon collection efficiency in this monitor has not been studied or even experimentally researched, what needs to be further determined.
6. For routine long term use, different types of ACF should be tested to select an ACF type with highest stability and more comfortable to be worn by individuals. Nevertheless, it is a regulatory requirement that for routine individual monitoring, detectors should be calibrated. In another study on ACF^37^, a module was studied which compensates the strong influence of temperature on the response of detectors with activated carbon or other ad/absorbents. The module is a hermetic volume made of polyethylene foil, through which radon diffuses and also stops ^220^Rn, as we also demonstrated before^20,37^. The module also protects the detector from humidity keeping its sensitivity to radon 7–9 times higher than that of commonly used radon detectors^20,37^.
7. Polycarbonate detector is flexible in size to be effectively used in different applications from small size (3 cm × 3 cm) as used in this study, in tissue-specific and energy-specific miniature dosimeter/spectrometers (3 cm × 3 cm)^33^, in multi-directional neutron spectrometry with polyethylene spheres (3 cm × 3 cm)^34^, in megasize in megasize radon monitors (40 cm × 80 cm)^21^, etc.
8. Our studies have provided succssful and promissing results for applying the dual-function individual radon monitor for miners in real radon working environments as well as for individual radon monitoring indoors of houses. The monitor can inherently filter radon and also particulates the efficieny of which needs to be determined. The monitor developed and its practical operation are on some basic fundamenal studies which has opened a new horizon for advanced fundamental research some of which are underway in our laboratory. Such studies may include enhancing the efficiency and the FAF value of the radon individual monitor by testing differrent types, thickness, brands, and dimensions of the ACF layer in particular for miners’ breathing confort; determining the ACF efficiency and the degree of filtering the inhaled radon progeny and particulates of different sizes; the effects of humidity and temperature on the response using diffeent ACF types; testing the monitor on respirators on miners in an operational mine environment and also individuals in a house; enhance the efficiency of PCTD further over a wider alpha registration energy range; apply other passive detectors like CR-39; study number of times an ACF filter can be charged and annealed to be reused again, etc.

## Conclusions

A novel dual-function passive radon monitor and protection respirator was invented, machine-made and studied for simulatious individual radon monitoring and protection agaist radon and particulates inhaled by individuals in particular by a miner in uranium and non-uranium mines. This is a breakthough invention based on determinig radon directly inhaled by an individual by using one PCTD/ACF detector in the monitor. A FAF value of 12.7 means that the monitor with PCTD/ACF, applying air suction or imhalation. provides12.7 times more sensitivity relative to PCTD/bare and when no air passes through. This value can be easily enhanced by further optimization studies.

The monitor provided successful and promissing results for individual radon monitoring and protection of miners as well as individual monitoring of residents in houses in only one device worn on the face. It is hoped that this novel individual radon monitor can better protect individuals against health hazards in particular in uranium and non-uranium mines and other possible applications in science and technology. This development has also opened a new horizon for extended fundamental and practical research which are being planned for future studies.

## Data Availability

The data on experimental results of this study are the data used for producing the graphs and all point data are reflected clearly on the graphs. Although the graphs clearly present the data used, if any person is interested to see the tabulated data, the data can be requested from the corresponding author upon request.
